# Machine learning-based real-time prediction of duodenal stump leakage from gastrectomy in gastric cancer patients

**DOI:** 10.3389/fsurg.2025.1550990

**Published:** 2025-05-06

**Authors:** Jae Hun Chung, Yushin Kim, Dongjun Lee, Dongwon Lim, Sun-Hwi Hwang, Si-Hak Lee, Woohwan Jung

**Affiliations:** ^1^ Division of Gastrointestinal Surgery, Department of Surgery, Pusan National University Yangsan Hospital, Yangsan, Republic of Korea; ^2^ Research Institute for Convergence of Biomedical Science and Technology, Pusan National University Yangsan Hospital, Yangsan, Republic of Korea; ^3^ Department of Surgery, School of Medicine, Pusan National University, Yangsan, Republic of Korea; ^4^ Department of Applied Artificial Intelligence (Major in Bio Artificial Intelligence), Hanyang University, Ansan, Republic of Korea; ^5^ Department of Artificial Intelligence, Hanyang University, Ansan, Republic of Korea

**Keywords:** duodenal stump leakage, gastrectomy, machine learning, gastric cancer, predictive modeling

## Abstract

**Purpose:**

This study aimed to develop a machine learning (ML) model for real-time prediction of duodenal stump leakage (DSL) following gastrectomy in patients with gastric cancer (GC) using a comprehensive set of clinical variables to improve postoperative outcomes and monitoring efficiency.

**Methods:**

A retrospective analysis was conducted on 1,107 patients with GC who underwent gastrectomy at Pusan National University Yangsan Hospital between 2019 and 2022. One hundred eighty-nine features were extracted from each patient record, including demographic data, preoperative comorbidities, and blood test outcomes from the subsequent seven postoperative days (POD). Six ML algorithms were evaluated: Logistic Regression (LR), K-nearest neighbors (KNN), Support Vector Machine (SVM), Random Forest (RF), Extreme Gradient Boosting (XGB), and Neural Network (NN). The models predicted DSL occurrence preoperatively and on POD 1, 2, 3, 5, and 7. Performance was assessed using the Area Under the Receiver Operating Characteristic Curve (AUROC) and Recall@K.

**Results:**

Among the 1,107 patients, 29 developed DSL. XGB demonstrated the highest AUROC score (0.880), followed by RF (0.858), LR (0.823), SVM (0.819), NN (0.753), and KNN (0.726). The RF achieved the best Recall@K score of 0.643. Including additional POD features improved the predictive performance, with the AUROC value increasing to 0.879 on POD 7. The confidence scores of the model indicated that the DSL predictions became more reliable over time.

**Conclusion:**

The study concluded that ML models, notably the XGB algorithm, can effectively predict DSL in real-time using comprehensive clinical data, enhancing the clinical decision-making process for GC patients.

## Introduction

Gastric cancer (GC) is the most prevalent malignancy of the upper gastrointestinal tract. It ranks fourth in terms of mortality and fifth in terms of the global incidence (1). The incidence rate of GC is the highest in Eastern Asia and Eastern Europe (2). While endoscopic submucosal dissection can be conducted selectively as a curative treatment option for early gastric cancer, gastrectomy remains the primary treatment for GC (3). However, this surgical approach is associated with significant risks of postoperative complications, including bleeding, bowel obstruction, duodenal stump leakage (DSL), anastomotic leakage, pancreatic fistula, pancreatitis, and delayed gastric emptying (4). Among these, DSL is one of the most severe complications owing to its high morbidity and mortality, with an incidence rate ranging from 1.6% to 5% and a mortality rate between 16% and 20% (5, 6). DSL can lead to a cascade of serious complications, including intraabdominal abscess, wound infection, necrosis or dehiscence, diffuse peritonitis, sepsis, malnutrition, fluid and electrolyte imbalances, dermatitis, acute cholecystitis, pancreatitis, abdominal bleeding, and pneumonia (5).

Despite its high potential risk (7), predicting DSL is a challenging problem owing to its low incidence and inherent patient safety issues, which limit prospective studies. Consequently, much of the existing research relies on retrospective analyses of medical records, employing statistical methods such as Student's *t*-test, chi-square test, and logistic regression to identify associated risk factors (8, 9). Previous studies have identified several risk factors for DSL, including inadequate duodenal stump closure, devascularization, cancer involvement at the resection line, inflamed duodenal wall, local hematoma, incorrect drain placement, multiple comorbidities, and postoperative duodenal distension (5, 10). The reconstruction method used after gastrectomy can also influence the risk of DSL. DSL occurs exclusively in patients undergoing Billroth II (BII) and Roux-en-Y (RY) reconstruction, both of which involve duodenal stump creation (11).

Machine learning (ML) methods have recently gained traction in the medical field, such as in cancer informatics and the prediction of postoperative complications (12, 13). Compared to the traditional statistical approaches, ML can manage a large number of variables effectively. Specifically, ML can capture and interpret complex interactions between input variables to make accurate outcome predictions (14).

But previous models for predicting postoperative complications have largely depended on static or early postoperative data, limiting their adaptability to the dynamic nature of clinical settings. To address these limitations, this study introduces a ML algorithms that integrates both preoperative variables and postoperative day (POD)-specific information. By incorporating time-sensitive clinical data, this approach aims to support real-time decision-making processes, offering a more responsive and tailored method for managing patient care in the postoperative period.

## Materials and methods

### Patients and data

**Figure 1:**
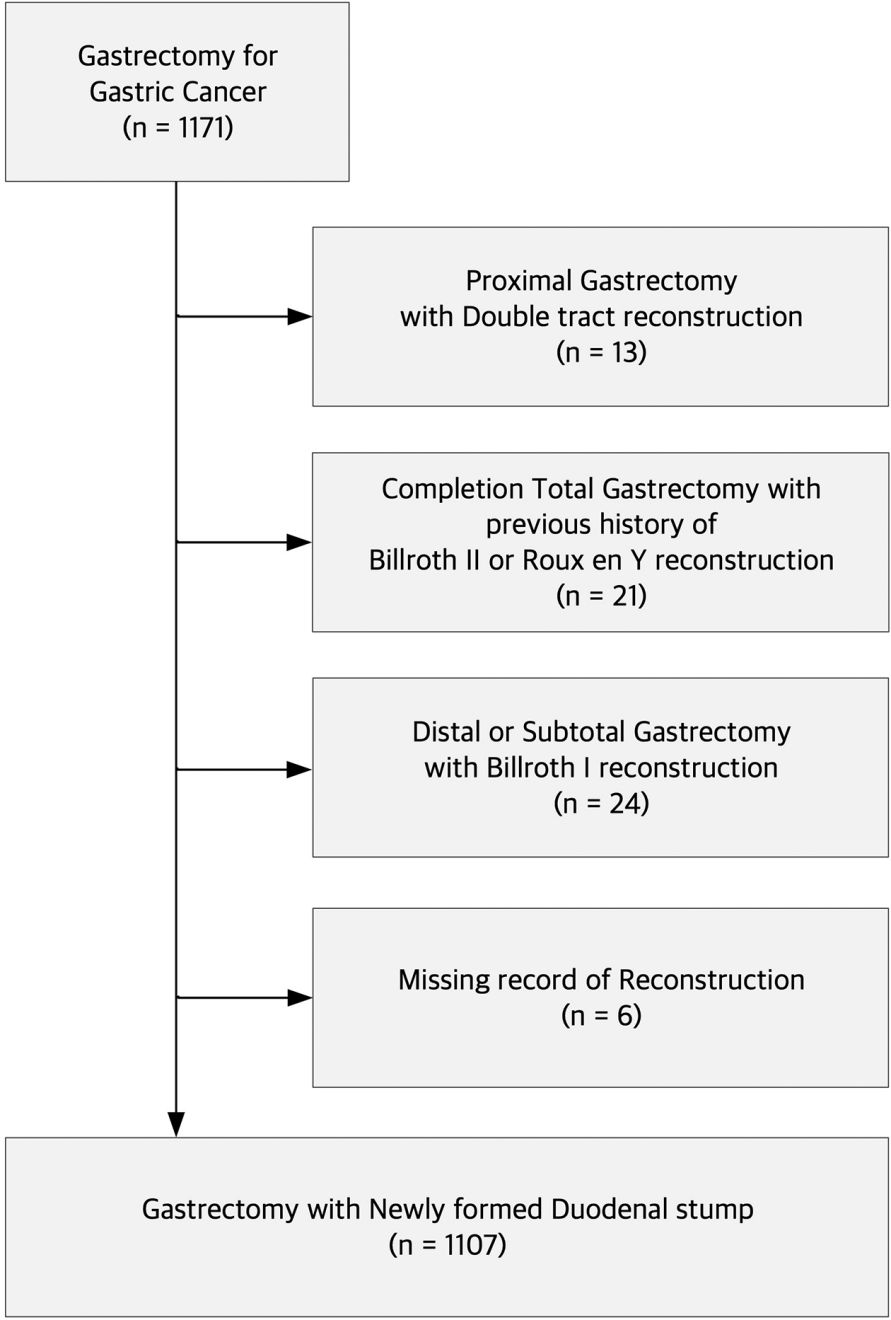
Consort diagram.

A total of 1,171 patients with gastric cancer underwent gastrectomy at Pusan National University Yangsan Hospital between 2019 and 2022. After excluding patients who underwent proximal gastrectomy with double-tract reconstruction (*n* = 13), completion total gastrectomy with a previous history of Billroth II or Roux-en-Y reconstruction (*n* = 21), distal or subtotal gastrectomy with Billroth I reconstruction (*n* = 24), and cases with missing reconstruction records (*n* = 6), we identified 1,107 patients who underwent gastrectomy with a newly formed duodenal stump (Figure 1). We collected retrospective data on clinicopathological and perioperative parameters to train the ML models for predicting DSL. An array of 189 features, including demographic data such as age, sex, body mass index (BMI), preoperative comorbidities, preoperative medical histories, and blood test outcomes from the subsequent seven PODs, were extracted from each patient record. Informed consent was not required for this study because of its retrospective nature and use of anonymous clinical data in the analysis. This study was approved by the Institutional Review Board of the Pusan National University Yangsan Hospital, Korea (IRB No. 05-2022-282).

### Predictor features and outcomes

**Figure 2:**
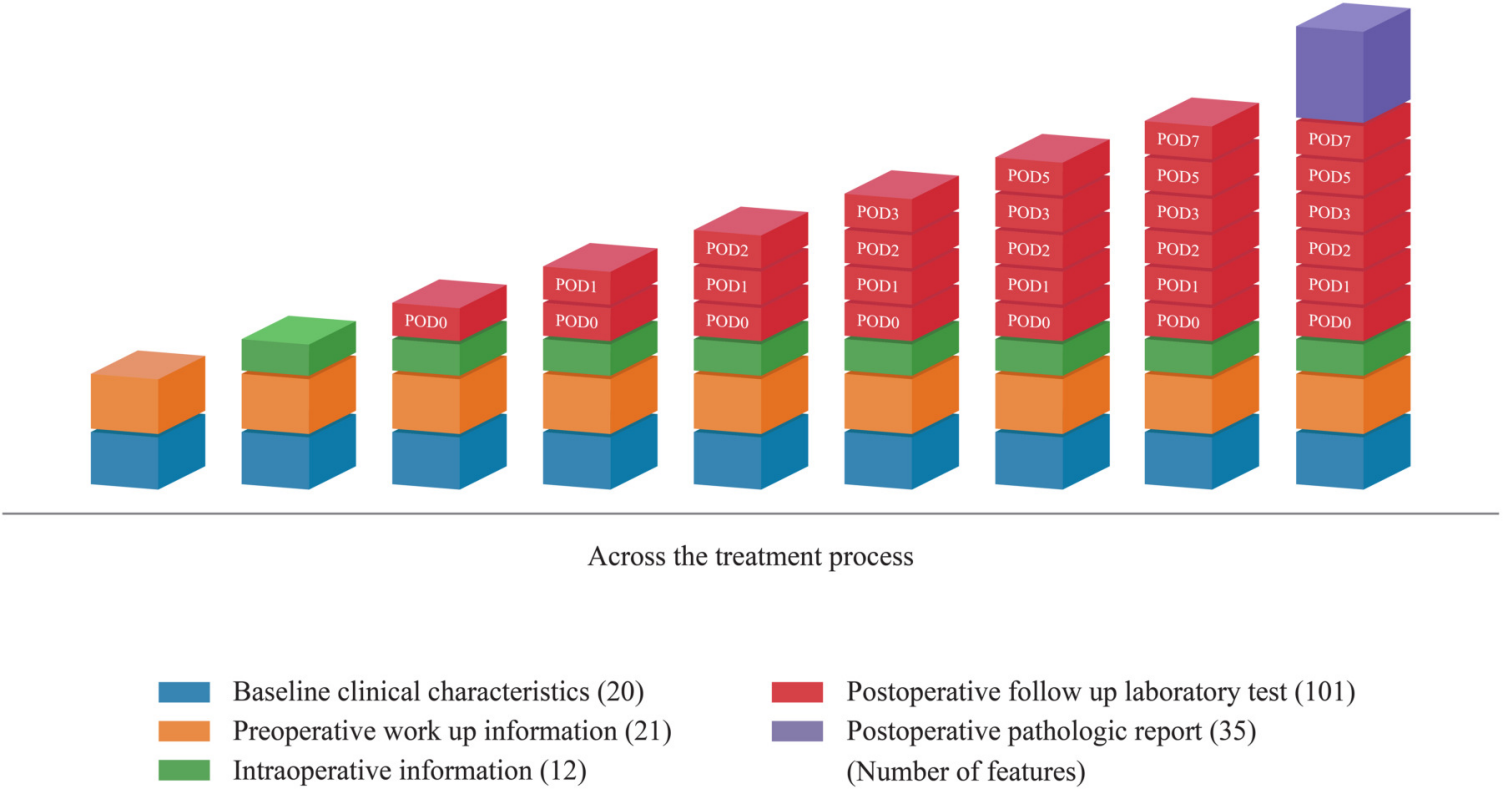
Distribution of 189 features across treatment phases.

The ML model predicted the occurrence of DSL at six specific time points: pre-operation, POD 0, POD 1, POD 2, POD 3, POD 5, and POD 7. Notably, Pre-operative predictions were made prior to gastric surgery, using only predictive features available at that time. These included demographic characteristics such as age and sex, the patient's medical history, and preoperative test results. For predictions on postoperative day (POD), additional features, including C-reactive protein (CRP), white blood cell (WBC) count, serum amylase, lipase, aspartate aminotransferase (AST), and alanine aminotransferase (ALT) obtained from blood test results up to that point, can be incorporated. Figure 2 provides a detailed visualization of the distribution of 189 features across treatment phases, categorized into baseline clinical characteristics, preoperative workup information, intraoperative information, postoperative laboratory tests, and pathology reports. Each category is represented as a stacked bar chart, with features distributed across specific time points: pre-operation, POD 0, POD 1, POD 2, POD 3, POD 5, and POD 7. Baseline clinical characteristics (20 features) include demographic and comorbidity data collected before surgery. Preoperative workup information (21 features) encompasses diagnostic imaging and laboratory results obtained prior to surgery. Intraoperative information (12 features) captures surgical details such as blood loss and anastomosis methods. Postoperative laboratory tests (101 features) include daily laboratory values from POD 0 to POD 7, such as CRP and WBC counts. Postoperative pathology reports (35 features) contain histopathological findings from resected specimens. The figure demonstrates how these features are cumulatively incorporated into the model at each prediction point. For example, on POD 3, the model uses preoperative features along with data from POD 1 to POD 3.

### Data preprocessing

Variability in data capture was observed across cohorts. This variation stemmed from some patients being discharged early or missing certain post-operative tests. To maintain a consistent data collection period across the patient cohort and prevent bias from incomplete data, we excluded 257 records representing patients with hospitalization durations of less than seven days. To address the issue of missing values, we applied an iterative imputation method from scikit-learn, which estimates missing values by creating a predictive model for each feature in a round-robin manner (15). At each step of the round-robin imputation, we use Bayesian Ridge regression since it is known to work well on data with small sample sizes (16). In addition, we standardized the numerical features to have a zero mean and unit variance.

To validate the model's performance, we compared four existing studies. Features that were not present in our dataset were excluded from this comparison. However, we supplemented the data with basic demographic information and underlying diseases, regardless of whether these factors had been reported in earlier studies, to ensure a comprehensive approach. This allowed us to confirm an empirical improvement in performance with our model. Table 1 presents a comparison of the features used or excluded in these existing studies.

**Table 1:** Comparison of features utilized to train machine learning.

Category	Features	Liu et al. (46)	Shao et al. (30)	Lee at al. (47)	Lee et al. (10)	This study
Baseline information	Age	O	O	O	O	O
	Sex	O	O	O	O	O
	BMI	O	O	O	O	O
	Underlying Diseases^a^	O	O	O	O	O
Clinical information	ASA score		O			O
	Smoking		O			O
	Surgery type		O			O
	Open Conversion		O			O
	Number of comorbidities				O	O
	Preoperative Hb	O	O			O
	Previous abdominal surgery		O			O
	Intraoperative bleeding	O	O			O
	Operation time		O			O
	Postoperative CRP (POD 1–5)	O				O
	Postoperative WBC (POD 1–5)	O				O
	CRP Reduction rate			O		O
	Combined resection		O			O
Tumor information	cTNM-T		O	O	O	O
	pTNM-T		O	O	O	O
	Maximum tumor size		O		O	O

BMI, body mass index; ASA, American Society of Anesthesiologists score; Hb, hemoglobin; CRP, C-reactive protein; POD, postoperative day; WBC, white blood cell.

^a^ Underly diseases included six categories: hypertension (HTN), diabetes mellitus (DM), cardiovascular disease, cerebrovascular disease, renal disease, and respiratory disease.

### Feature selection

This study aims to enhance DSL prediction following gastrectomy in patients with gastric cancer. We hypothesized that leveraging a comprehensive set of features could significantly improve the performance of machine learning models in predicting DSL. By incorporating a number of features, including daily postoperative data, the goal was to achieve a more accurate representation of a patient's health status, leading to better predictive outcomes. To assess the impact of feature quantity on model performance, we compared the prediction accuracy across different sets of features.

The selected features include nine basic features (i.e., 3 demographic informations; age, sex, BMI and underlying diseases; HTN, DM, cardiovascular disease, cerebrovascular disease, renal disease, respiratory disease) to ensure a fair comparison (Table 1). The remaining features, ranging from one to 151, were randomly selected, resulting in 10–160 features for each experiment. Each experiment was repeated ten times to estimate the mean performance and variance.

### Machine learning algorithms

We evaluated the effectiveness of our method using six widely adopted machine learning algorithms. Each algorithm can calculate the probability of a patient developing DSL, which we refer to as the model's confidence level. The algorithms were implemented in Python 3.11, with XGB using the xgboost 1.7.5 library, while the remaining models were implemented using the scikit-learn library.

(1) Logistic regression (LR): A classification model that estimates the probability of binary outcomes based on a set of predictor variables. We used the C parameter set to 0.01, which controls the regularization strength. We applied L2 regularization to prevent overfitting by penalizing large coefficients. The L-BFGS solver was applied for optimization.
(2) K-nearest neighbors (KNN): This approach classifies new data points by considering their k-nearest instances in the training data, often using a majority voting scheme. We set the K to 9, which considers the 9 nearest neighbors to make predictions.
(3) Support vector machine (SVM): An algorithm that classifies data by determining the optimal hyperplane, maximizing the margin between two classes (17). We used the C parameter set to 1, which controls the regularization strength. A Radial Basis Function (RBF) kernel was applied to handle non-linear relationships in the data.
(4) Random forest (RF): An ensemble learning method that uses multiple decision trees to mitigate overfitting and effectively handle large datasets. We limited the depth of each tree to 8. Each tree was trained on a random sample containing 50% of the data, and the model consisted of 100 decision trees.
(5) Extreme gradient boosting (XGB): An optimized distributed gradient boosting algorithm designed to handle complex interactions among features (18). We used a learning rate of 0.05 to control the step size at each iteration. The depth of each tree was set to 6, and the model was configured to grow 100 trees in parallel. Additionally, the model used a subsample ratio of 33% of the data for each tree to introduce variability. We applied the lossguide grow policy to prioritize splitting nodes with the highest loss reduction.
(6) Neural network (NN): This algorithm denotes a feed-forward neural network. We used two hidden layers, each containing 150 neurons. The learning rate was set to adaptive, allowing the model to adjust the rate based on performance during training. The model was trained for a maximum of 1,000 iterations, and the L-BFGS solver was applied for optimization. To prevent overfitting, the number of hidden layers in the neural network was limited to two. When using deeper architectures, techniques such as dropout (19) and batch normalization (20) can be employed to mitigate overfitting.

Specifically, LR is often favored for its interpretability, which is important in clinical settings (21). SVM and RF are known for handling high-dimensional datasets. Thus, they were the best performers in an extensive empirical evaluation with 18 ML algorithm families (22). Since our data has 189 features, we selected those algorithms. Gradient boosting algorithms, such as XGB, have gained prominence due to their ability to model complex interactions within the data while maintaining high accuracy (23). In addition, we incorporated NN, which was not considered in earlier works since it is one of the most widely used machine learning models today (24).

### Cross-validation

**Figure 3:**
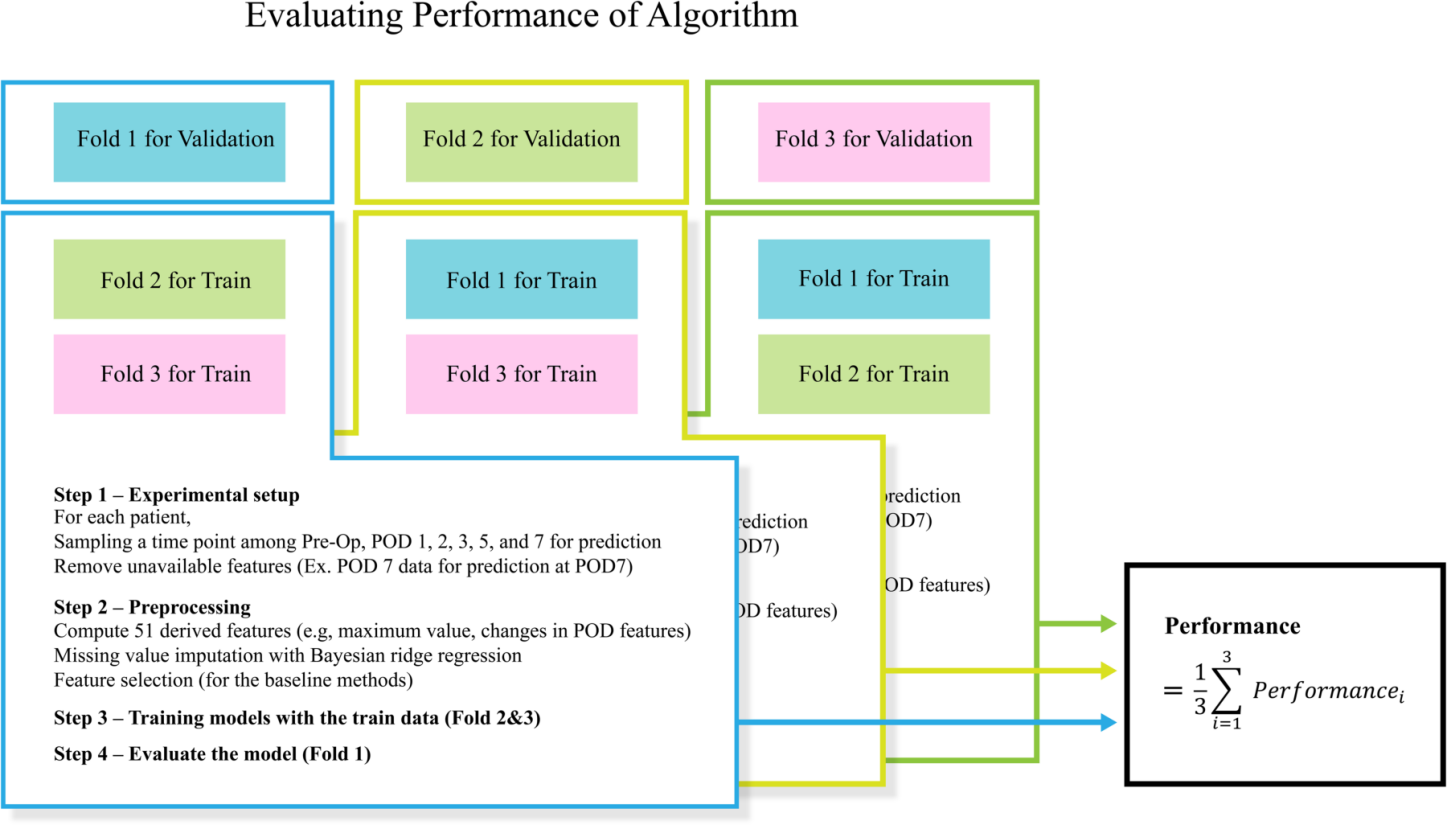
3-fold cross-validation of performance.

Cross-validation is used to evaluate the performance of models by testing them on different subsets of the data, ensuring robustness and generalizability. It provides a more reliable estimate of a model's performance than a single train-test split. In this study, we utilized 3-fold cross-validation (Figure 3). It divides the dataset into three groups or folds. During evaluation, three iterations are performed, with each iteration using two folds for training and the remaining fold for validation. The average scores across these iterations are reported, providing a comprehensive assessment of the model's performance.

### Evaluation measures for ML algorithms

We used AUROC and Recall@K as evaluation measures. The AUROC is a performance metric used to evaluate binary classification models. It measures how well a model can distinguish between two classes by calculating the area under the ROC curve, which plots the true positive rate (sensitivity) against the false positive rate (1—specificity) at different classification thresholds. The ROC curve provides a comprehensive threshold-independent view of the model's performance. An AUROC value of 1 represents perfect classification, while a value of 0.5 indicates the model performs no better than random chance. Recall@K measures the model's ability to identify the K most at-risk patients, which is especially useful in imbalanced datasets with a few positive cases (25). We set K to 36, corresponding to approximately 10% of the test set in each cross-validation fold. Recall@K is defined as:

$$\mathrm{Recall}@K=\;\frac{\left(\mathrm{Number}\;\mathrm{of}\;\mathrm{DSL}\;\mathrm{patients}\;\mathrm{in}\;\mathrm{the}\;\mathrm{top}\;K\;\mathrm{predicted}\;\mathrm{patients}\right)}{\left(\mathrm{Number}\;\mathrm{of}\;\mathrm{DSL}\;\mathrm{patients}\right)}$$

This metric assesses how well the model prioritizes patients at high risk of developing DSL, helping to guide targeted interventions. Specifically, Recall@36 represents the percentage of DSL cases captured when focusing on the top 10% of patients ranked by each model.

### Observation of model confidence

We analyze the confidence scores generated by the machine learning model, representing the predicted probability of DSL. Specifically, we focused on observing the progression of these confidence scores over time, particularly as the number of postoperative days (POD) increased. To accomplish this, we sampled 10 patients each from the DSL and non-DSL groups and calculated their respective average confidence scores for each predictive day.

This analysis provides clinically relevant insights, indicating when the model's predictions become reliable. From a machine learning perspective, it demonstrates how effectively our model leverages accumulated post-operative data over time to enhance its predictive accuracy.

## Results

### Demographic and clinicopathological characteristics of the patients

In this cohort, DSL was diagnosed in 29 individuals. Table 2 compares the clinicopathological characteristics of 29 patients with DSL and 1,078 patients without DSL. The characteristics compared age; sex distribution; BMI; ASA score; underlying diseases such as hypertension, diabetes mellitus, dyslipidemia, cardiovascular disease, cerebrovascular disease, nephrology, and respiratory diseases; and TNM stages (T, N, and M) based on the 8th edition of the American Joint Committee on Cancer TNM classification. The *p*-values indicate statistical significance, with notable differences observed in sex distribution and the presence of respiratory diseases between the two groups.

**Table 2:** Comparison of clinicopathological characteristics of patients with DSL.

Clinicopathologic characteristics	No DSL	DSL	*p*-value
	*n* = 1,078	*n* = 29	
Age (years)	64.13 ± 0.34	64.55 ± 2.01	0.840
Sex (male/female)	721/357	26/3	0.010
BMI (kg/m^2^)	25.96 ± 1.87	25.17 ± 0.53	0.945
ASA score			0.521
1	309	7	
2	654	21	
3	112	1	
4	3	0	
Underlying disease			
Hypertension	370	9	0.713
Diabetes mellitus	193	8	0.406
Dyslipidemia	121	4	0.666
Cardiovascular disease	91	2	0.767
Cerebrovascular disease	61	1	0.609
Nephrology disease	34	2	0.262
Respiratory disease	5	2	0.033
T stage^a^			0.886
1	705	21	
2	95	3	
3	127	2	
4	151	3	
N stage^a^			0.394
0	791	24	
1	98	2	
2	91	0	
3	98	3	
M stage^a^			0.471
0	1,059	29	
1	19	0	

BMI, body mass index; ASA, American Society of Anesthesiologists score.

^a^ According to the 8th edition of the American Joint Committee on Cancer TNM classification.

### Predictive performance

**Figure 4:**
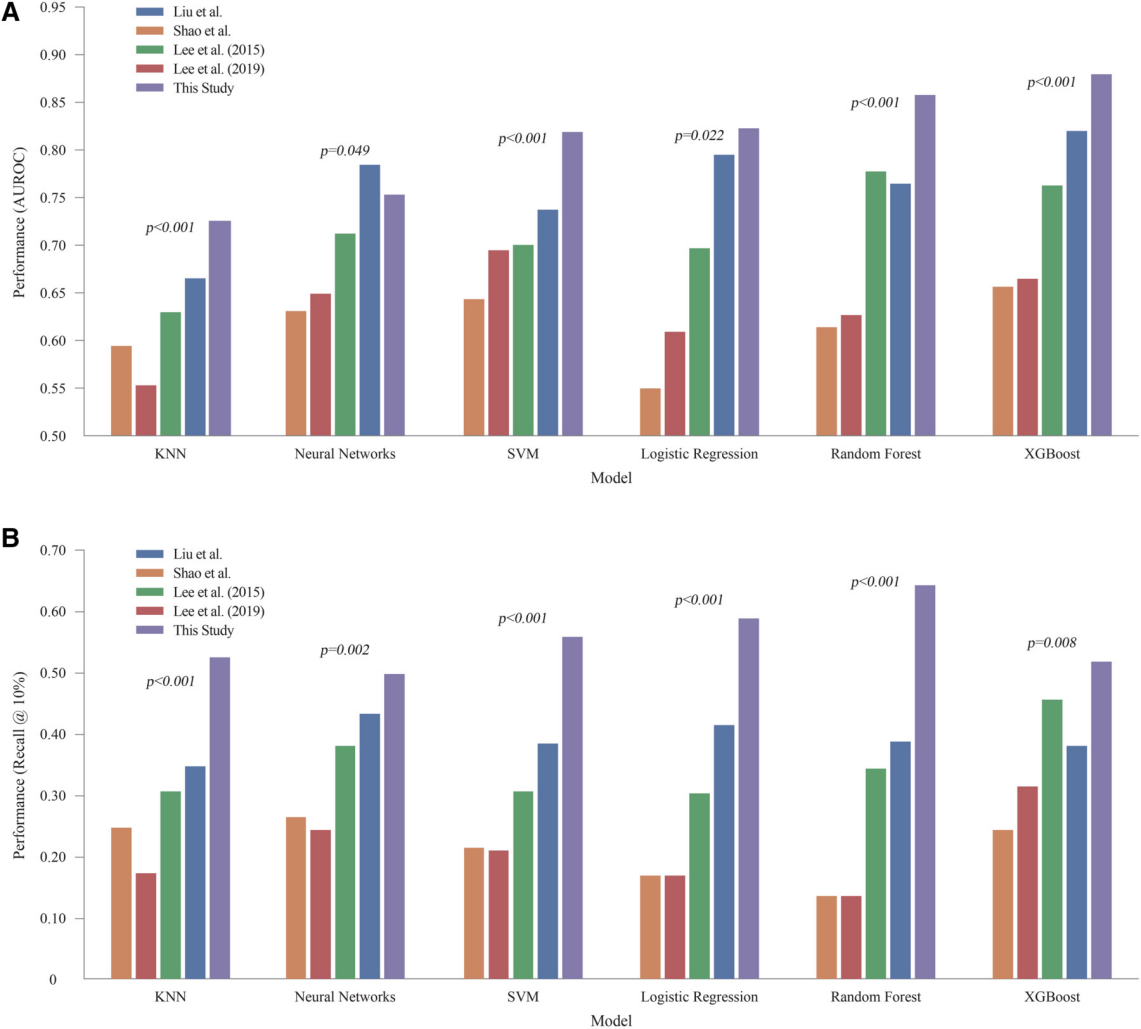
Comprehensive performance of all models across all prediction time **(A)** AUROC of machine learning models. **(B)** Recall @ 10%. LR, logistic regression; KNN, K-nearest neighbors; SVM, support vector machine; RF, random forest; XGB, extreme gradient boosting; NN, neural network.

Table 3 and Figure 3 present the performance of all compared models across all prediction time points. The comparison revealed that our study achieved the highest AUROC across five machine learning models except NN. Specifically, the XGB model demonstrated the highest AUROC score of 0.880, followed by RF at 0.858, LR at 0.823, SVM at 0.819, NN at 0.753, and KNN at 0.7256 (Figure 4A). The RF model achieved the best recall at 10% with a score of 0.643, indicating that it was the most successful in correctly identifying the top 10% of patients at risk of DSL. The LR scored 0.589 in the recall at 10%, followed by SVM at 0.559, KNN at 0.526, XGB at 0.519, and NN at 0.498 (Figure 4B). Upon comparing the highest and second-best AUROC values across all models, excluding NN, our study results were significantly superior, as evidenced by the p-values: KNN (*p* *<* *0.001*), SVM (*p* *<* *0.001*), LR (*p* *=* *0.022*), RF (*p* *<* *0.001*), and XGB (*p* *<* *0.001*). In the analysis of recall at 10%, our study also demonstrated statistically significant higher values across all models compared to the second-best recall at 10%, with the respective *p*-values: KNN (*p* *<* *0.001*), NN (*p* *=* *0.002*), SVM (*p* *<* *0.001*), LR (*p* *<* *0.001*), RF (*p* *<* *0.001*), and XGB(*p* *=* *0.008*).

**Table 3:** Overall predictive performance for each study.

Study	# Features	Model	AUROC	Recall @K^a^
Liu et al. (46)	29	LR	0.796	0.415
		KNN	0.667	0.348
		SVM	0.739	0.385
		RF	0.766	0.388
		XGB	0.822	0.381
		NN	0.786	0.433
Shao et al. (30)	26	LR	0.551	0.170
		KNN	0.596	0.248
		SVM	0.644	0.215
		RF	0.615	0.137
		XGB	0.658	0.244
		NN	0.633	0.265
Lee et al. (10)	15	LR	0.698	0.304
		KNN	0.631	0.307
		SVM	0.702	0.307
		RF	0.779	0.344
		XGB	0.764	0.456
		NN	0.714	0.381
Lee et al. (47)	14	LR	0.610	0.170
		KNN	0.554	0.174
		SVM	0.696	0.211
		RF	0.628	0.137
		XGB	0.666	0.315
		NN	0.650	0.244
This study	189	LR	0.823	0.589
		KNN	0.726	0.526
		SVM	0.819	0.559
		RF	0.858	0.643
		XGB	0.880	0.519
		NN	0.753	0.498

LR, logistic regression; KNN, K-nearest neighbors; SVM, support vector machine; RF, random forest; XGB, extreme gradient boosting; NN, neural network.

^a^ K = 36 indicates the top 10% of patients recommended by each ML model.

.

### Experiments on the number of features

**Figure 5:**
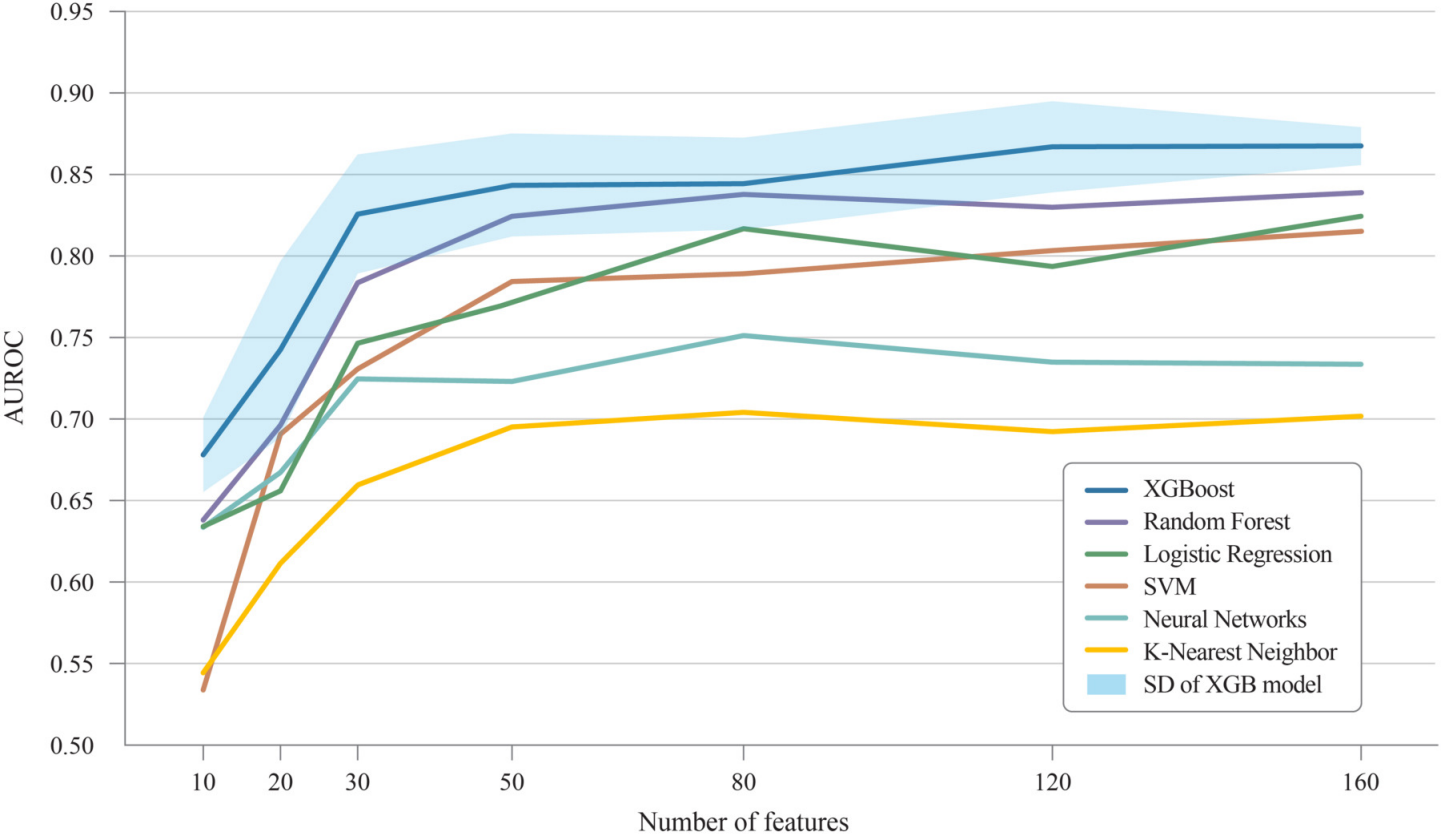
Improvement of AUROC scores with additional features. AUROC, area under receiver operating characteristic curve; SD, Standard deviation.

Figure 5 demonstrates the change in AUROC values as the number of features increases across different ML models. XGB showed the highest improvement, with an AUROC of 0.678 for 10 features, reaching 0.867 with 120 features. Similarly, other models like SVM, RF, and LR also showed significant improvements as more features were included. The SVM model, for instance, rose from 0.534 with 10 features to 0.803 with 120 features. The RF model increased from 0.638 to 0.830 over the same range. The standard deviations of the AUROC values across the XGB model with varying feature sets were ±0.023, ± 0.054, ± 0.036, ± 0.032, ± 0.028, ± 0.028, and ±0.012, respectively. This indicates that increasing the number of features leads to reduced variance, thereby enhancing the reliability of the model's predictions.

### Effectiveness of POD information

**Figure 6:**
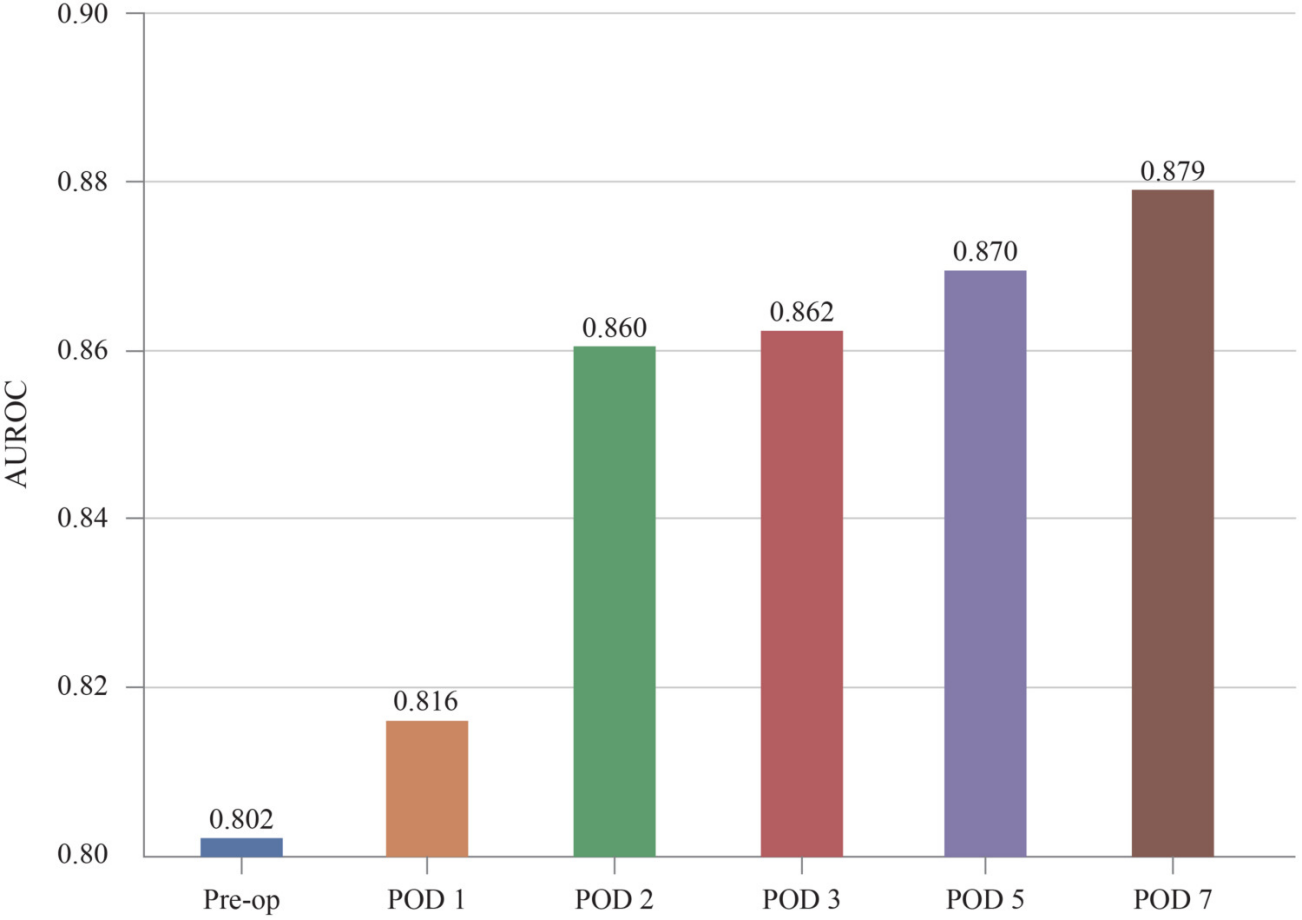
Increase of predictive performance as more POD information is provided. AUROC, area under receiver operating characteristic curve; POD, postoperative day.

The models predicted DSL occurrence preoperatively and on POD 0, 1, 2, 3, 5, and 7. Patients who were discharged before each prediction time point were excluded from the predictions. As a result, 1,107 patients are in the dataset until POD3. while 1,102 and 850 patients remain for POD5 and POD7 predictions, respectively. Figure 6 illustrates the evolution of the predictive performance of our XGB model as POD information was progressively included. The analysis began with a model that used only pre-operative information, achieving an AUROC of 0.802. Successive POD information was gradually introduced for prediction from POD 1 to POD 7. This series of experiments simulated real-time prediction scenarios, capturing the progression from patients who had just undergone surgery to those who had recovered over several days. Each bar in the graph represents the area under the ROC curve for each stage of the analysis. The predictive performance improved from 0.802 to 0.879 with the availability of additional POD information.

### Evolution of confidence score

**Figure 7:**
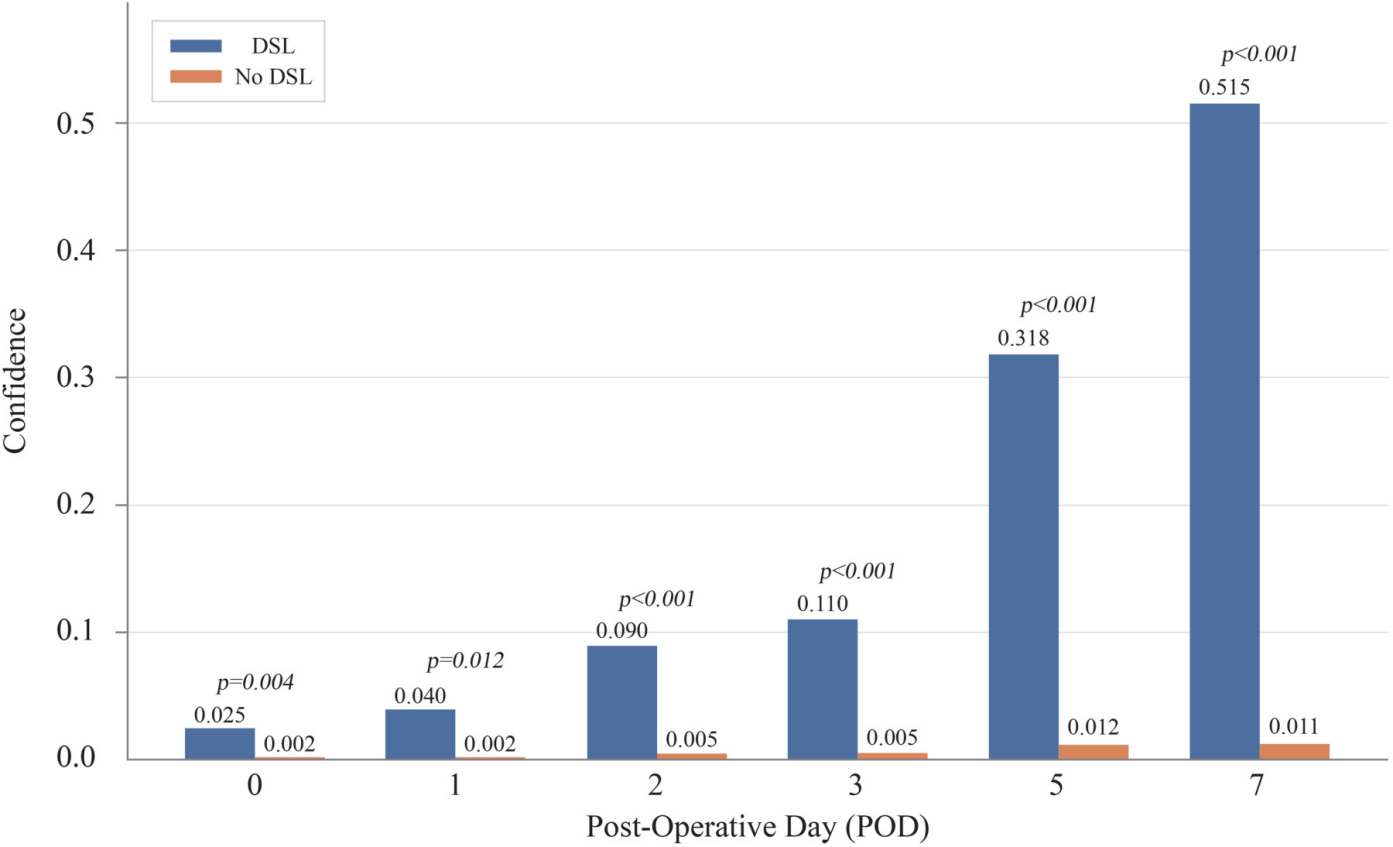
Evolution of confidence score by time. DSL, duodenal stump leakage.

Figure 7 illustrates the average confidence scores for cases with and without DSL at different prediction times. As more POD information was added, the average confidence score for DSL cases increased, eventually reaching 0.515 by POD 7, while the score for non-DSL cases remained low at only 0.011. The gap between the confidence scores for DSL and non-DSL cases widened over time, reinforcing our hypothesis that incorporating POD information improves the model's ability to distinguish between these two groups. For reference, the confidence scores and *p*-values at each POD were: POD 0 (DSL: 0.025, No DSL: 0.002, *p* *=* *0.004*), POD 1 (DSL: 0.040, No DSL: 0.002, *p* *=* *0.012*), POD 2 (DSL: 0.090, No DSL: 0.005, *p* *<* *0.001*), POD 3 (DSL: 0.110, No DSL: 0.005, *p* *<* *0.001*), POD 5 (DSL: 0.318, No DSL: 0.012, *p* *<* *0.001*), and POD 7 (DSL: 0.515, No DSL: 0.011, *p* *<* *0.001*).

## Discussion

DSL is one of the most severe complications following gastrectomy, with high morbidity and mortality rates. However, due to the low incidence of DSL, conducting large-scale studies or drawing statistically significant conclusions is challenging. Our study found that the high generalization performance of ML algorithms allowed for the early-stage risk detection of DSL. Therefore, the use of ML models for DSL predictions is crucial.

Statistics are typically used to draw inferences and derive scientific insights from data collected from a given population (26). In contrast, ML is particularly useful for making predictions when working with a larger set of variables and considering the complex relationships between input and output variables (27, 28). This is particularly valuable in clinical settings where the ability to predict outcomes in real-time can significantly enhance patient care. Therefore, this study focused on leveraging the advantages of ML to predict rare but significant occurrences such as DSL. Despite its low incidence, the accurate prediction of DSL is crucial for improving patients’ postoperative outcomes and monitoring efficiency.

Several studies have developed models using various machine-learning algorithms to predict complications after gastric surgery. For instance, Hong QQ et al. employed LASSO regression, Random Forest(RF), and Neural Network(NN) models to predict the incidence of complications on the third postoperative day after radical gastrectomy (29). Additionally, Shao et al. utilized Extreme Gradient Boosting(XGB) to develop a model assessing the risk of anastomotic leakage following gastrectomy (30). These prior studies offer valuable insights for surgeons to predict complications preoperatively or immediately postoperatively, thereby enhancing clinical practice. However, in the real-world clinical setting, where patient conditions change in real-time and blood tests vary throughout the postoperative hospital stay, these research outcomes may not be suitable for real-time prediction of complications. For practical application in clinical settings, prediction models must be responsive to daily fluctuations in patient conditions and the results of ongoing follow-up tests.

We observed that the models with the highest AUROC and those with the highest Recall@K were not always the same (Table 3 and Figure 4). AUROC provides a comprehensive, threshold-independent assessment of a model's performance across all potential decision thresholds, making it useful for evaluating the overall stability and effectiveness of the algorithm. On the other hand, Recall@K is more context-specific, measuring the model's ability to capture a high percentage of true DSL cases within the top K patients, which is particularly valuable when resources are limited, and a hospital needs to focus on a fixed number of high-risk patients. Given the variability in practical settings where the value of K can be adjusted based on clinical needs, it is important to use both metrics. While AUROC indicates the general robustness of a model, Recall@K allows for more targeted decision-making. In real-world applications, choosing an algorithm based on its performance in Recall@K, tailored to the desired K, could lead to more effective patient management strategies.

However, the results presented in Table 3 and Figure 4 demonstrate the effectiveness of our strategy, which utilizes all the available features. Except for experiments with neural networks, our strategy outperforms existing methods that rely on features that are selectively chosen by researchers. Neural networks tend to overfit data (25). Therefore, it is recommended to reduce the number of input features to prevent this issue. Chen et al. emphasized the importance of feature selection, particularly for datasets with various variables and features (31). Their study showed that proper feature selection helps eliminate less critical variables, thereby enhancing the accuracy and performance of the classification models. This underscores the advantages of careful feature selection. A model trained with a well-curated set of features generally outperforms a model with a comparable number of randomly selected features. Nevertheless, our primary focus was on the benefits of applying ML to comprehensive feature sets.

As shown in Figure 5, regardless of the number of input features, the accuracy of the XGB algorithm consistently surpassed that of the other ML methods, indicating that XGB is suitable for real-time DSL prediction. Although XGB's effectiveness in tabular data has been highlighted in various studies (32, 33), our findings suggest that it is particularly effective in processing POD information, even with many missing values. This characteristic, combined with our extensive feature set, likely explains the robust performance in predicting DSL. Because the performance of ML models improves as the number of input features grows, the accuracy of predictions tends to improve over time as more postoperative test results become available.

Figure 6 shows that the accuracy improved notably when results from POD 2 were included. As more postoperative blood tests are conducted, the volume of data encompassing a wider range of features gradually increases. This temporal data enrichment, as depicted in Figures 5–7, leads to enhanced predictive accuracy for DSL, evidenced by the rising AUROC values. Along with this trend, a reduction in the standard deviation was observed, corresponding to the increasing postoperative variables. For real-time prediction of patient complications, acknowledging and incorporating the expanding nature of the dataset throughout the postoperative period is essential. The findings from this study indicate that, with the inclusion of more features, models like XGB, SVM, RF, and LR not only improved their performance but also displayed enhanced robustness. Therefore, these models are suitable for real-time prediction of complications and can help provide a nuanced understanding of a patient's evolving condition.

Three key insights can be derived from Figure 7. First, the overall confidence scores appear relatively low, largely due to significant class imbalance from the sparsity of DSL cases. However, these low scores do not imply poor predictive performance, as evidenced by the high area under the AUROC scores. If needed, techniques such as oversampling can be used to adjust the confidence scores and address class imbalance (34). Secondly, contrary to expectations, the model did not decrease its confidence scores for non-DSL records as more POD information was introduced. Instead, these scores increased, but at a slower pace compared to the DSL cases. This widening gap between confidence scores for DSL and non-DSL cases suggests that adding more POD information enhances the model's ability to differentiate between the two classes, supporting our hypothesis. Finally, an interesting inflection point was observed when the model received POD 5 information. The sharp increase in confidence scores for DSL cases—reaching 0.318 by POD 5 and 0.515 by POD 7—indicates that these time points may contain particularly crucial clinical information with significance. These results support the previously identified timing of DSL incidence, which typically occurs between 5 and 10 days post-surgery, as reported in the existing literature (35).

This analysis offers valuable insights into how the confidence of the XGB model in its predictions evolves over time, highlighting the significant role of POD information in enhancing the model's ability to differentiate between patients who will develop DSL and those who will not.

**Figure 8:**
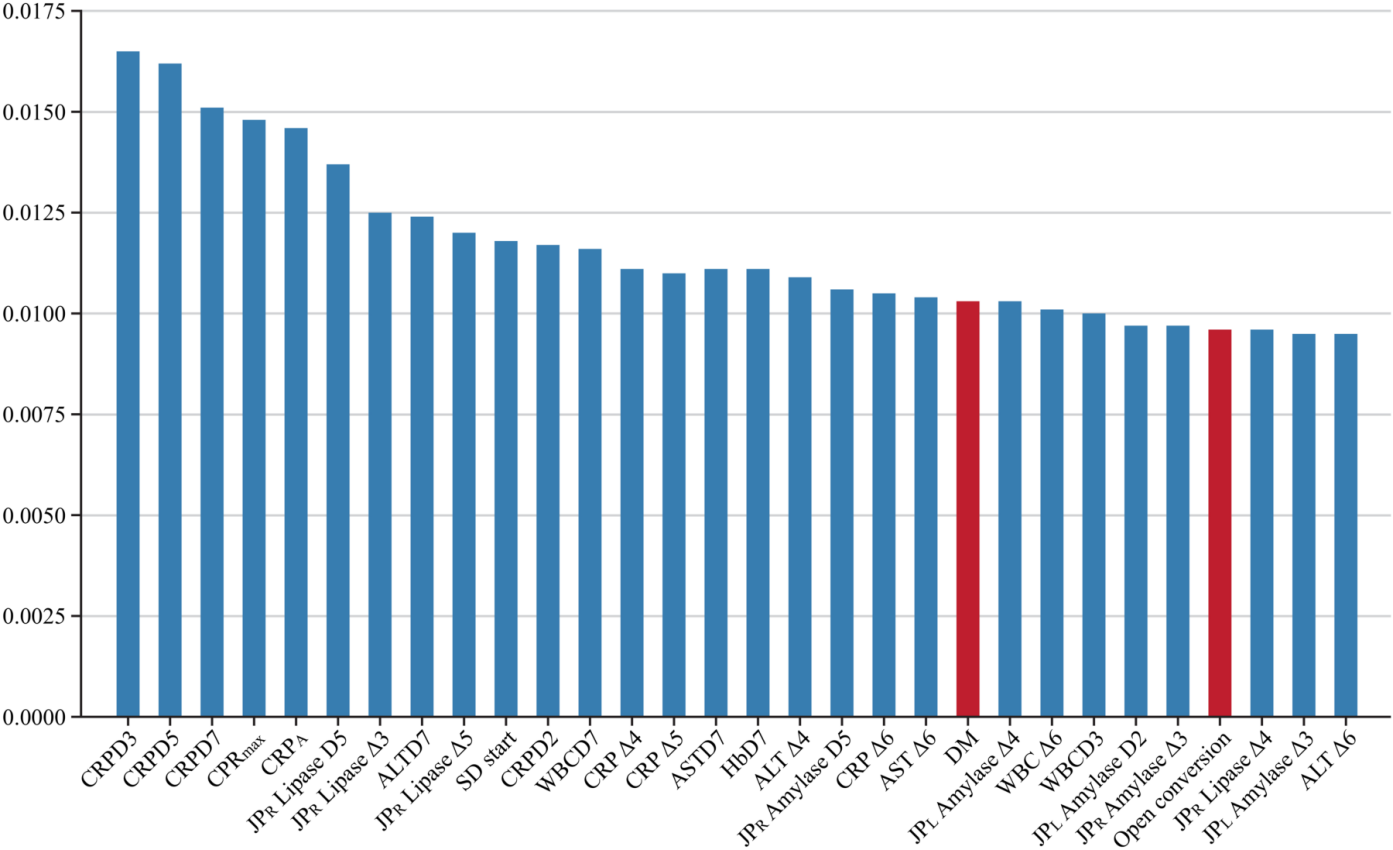
Top 30 important features. CRPDn, CRP on postoperative day n; CRP *Δ*n, rate of decrease in CRP levels on postoperative day n; HbDn, Hemoglobin on postoperative day n; ASTDn, AST on postoperative day n; ALTDn, ALT on postoperative day n; JP_L or R_ Amylase or Lipase *Δ*n, rate of decrease in amylase or lipase levels on postoperative day n; WBCDn, WBC on postoperative day n; WBC *Δ*n, rate of decrease in WBC levels on postoperative day n; SD, Soft diet.

Most previous studies on DSL have identified pre-operative risk factors, suggesting that they might be among the highly important features in this study. We anticipated that several factors from earlier research, such as gastric outlet obstruction, liver cirrhosis, and cardiovascular disease, would be included among the important features; however, our results differed (10, 36). According to the findings of this study, among the top 30 important features were identified (Figure 8). Clinically, this is a significant finding. Previous studies have identified factors such as non-reinforcement of the duodenal stump, BMI (≥ 24 kg/m²), and elevated preoperative CRP levels as risk factors for DSL. Although no studies have specifically identified DM as a risk factor for DSL, it has been noted in prior research as a systemic condition that impairs recovery after bowel anastomosis or injury (37). Among the top features predicting DSL, DM is the only factor reflecting the preoperative condition of the patient. Therefore, further studies are needed to investigate the correlation between DM and DSL. Previous *in vivo* studies have shown that diabetes leads to alterations in cellular components involved in the early phase of repair of intestinal anastomoses (38). This impairment is primarily due to chronic hyperglycemia, which damages vasculature and hinders proper blood perfusion, thereby disrupting the healing processes (39). Moreover, the other top features, excluding the day of soft diet started and DM, were predominantly related to two main clinical aspects: the patient's systemic inflammatory state and the detection of leaking pancreatic juice. For instance, CRP levels and WBC counts reflect the body's inflammatory response, which can indicate complications such as infections or an ongoing inflammatory process at the surgical site. Elevated lipase and amylase levels in the drainage fluid are crucial for detecting leakage from the pancreas, which is a direct contributor to DSL. These biomarkers are essential for early detection and timely intervention, underscoring the importance of continuous monitoring during the postoperative period. These clinical insights demonstrate the practical application of ML in identifying crucial predictive features. Unlike traditional statistical methods, ML is not solely focused on the relationships among variables but on their direct impact on patient outcomes. This emphasizes the potential of ML to enhance clinical decision-making by pinpointing the most relevant factors that contribute to postoperative complications (40).

XGB relies on multiple decision trees, allowing us to measure the importance of a feature by counting the number of times it is used to split the data across all trees. Figure 8 illustrates the proportion of features utilized by XGB. Several clinical studies using machine learning have extracted a subset of features from their databases as the optimal feature set for predicting specific issues. According to a systematic review, earlier studies on AI prediction of surgical complications analyzed 16,193 features (41). However, as commonly recognized in the machine learning community, the optimal feature set can vary depending on the size of the dataset (42, 43). This variability makes it challenging to expect that a feature set derived from one research study using data from a specific hospital will also be optimal when applied to other hospitals. In this study, we empirically demonstrate that the XGB (18) and SVM (17) algorithms achieve better performance when trained on a larger number of features compared to using feature sets identified in previous studies. Additionally, we show that these algorithms maintain stable performance despite variations in the number of features. These results suggest that our approach could be effective when utilizing data from different institutions to train DSL prediction models, offering greater generalization and adaptability across various clinical settings.

Moving beyond simple complication prediction, actionable clinical scenarios can be envisioned for applying this model. Evidence on intraoperative drain placement remains controversial. Weindelmayer et al. found that avoiding drains after gastrectomy for gastric cancer may increase the risk of postoperative invasive procedures, supporting routine drain use (44). In contrast, Lim et al. reported limited benefits of prophylactic drainage in preventing major intra-abdominal complications, aligning with the growing preference for drain omission under enhanced recovery after surgery (ERAS) protocols (45). Our predictive model aims to resolve these conflicting approaches by providing real-time risk assessment for DSL, a severe complication linked to fluid collections. By guiding surgeons on whether to place a drain during surgery and when to remove it postoperatively, the model enhances safety while supporting ERAS principles, potentially improving recovery outcomes and standardizing decision-making.

To facilitate implementation in clinical practice, we propose integrating the ML model directly into the hospital's Electronic Medical Record system (EMR). By doing so, all postoperative patient monitoring results can be automatically consolidated and analyzed by the ML model. This setup would enable immediate prediction and notification to medical staff when the probability of complications spikes. Implementing such a system would significantly aid in the early diagnosis and prompt additional intervention or image workup of patient complications, ultimately improving patient outcomes in terms of both mortality and morbidity. This would be a vital integration into conventional EMR systems, reinforcing the practical implications and benefits of our predictive model in clinical workflows.

This study is the first to develop a real-time prediction model for DSL following gastrectomy in patients with GC. However, it has several limitations. One limitation of the ML approach is its inability to explain all correlations between features. Additionally, the algorithm was trained using retrospective clinical data from a single center, which implies that it may not be generalizable to other medical institutions. Applying this model to other settings would likely require retraining with their specific datasets to ensure accuracy and reliability. To enhance generalizability, future work should focus on external validation using multi-institutional datasets from diverse geographical regions and clinical settings. Additionally, we recommend implementing transfer learning approaches that allow the model to be fine-tuned with smaller datasets from new institutions, potentially improving adaptability while maintaining predictive performance. Despite these limitations, our comprehensive feature selection approach and temporal modeling strategy provide a framework that could be replicated across institutions, even if specific model parameters require adjustment.

## Conclusion

According to this study, handling high-dimensional data with ML improves predictive efficacy and enhances robustness in the clinical field. A real-time predictive model will assist gastrointestinal surgeons in accurately predicting DSL after gastrectomy, allowing them to make informed decisions about adding additional follow-up tests, implementing more careful patient management, and determining the appropriate timing for discharge.

## Data Availability

Although the study received IRB approval with a waiver of informed consent, there was no separate review regarding external sharing of the data. Therefore, the raw data cannot be made publicly available due to institutional policy. Further requests or queries can be directed to the corresponding author.

## References

[1] Bray F Ferlay J Soerjomataram I Siegel RL Torre LA Jemal A. Global cancer statistics 2018: GLOBOCAN estimates of incidence and mortality worldwide for 36 cancers in 185 countries. CA Cancer J Clin. (2018) 68(6):394–424. 10.3322/caac.21492 30207593

[2] Ferlay J Colombet M Soerjomataram I Parkin DM Pineros M Znaor A Cancer statistics for the year 2020: an overview. Int J Cancer. (2021) 149(4):778–89. 10.1002/ijc.33588 33818764

[3] Ramos M Pereira MA Barchi LC Yagi OK Dias AR Szor DJ Duodenal fistula: the most lethal surgical complication in a case series of radical gastrectomy. Int J Surg. (2018) 53:366–70. 10.1016/j.ijsu.2018.03.082 29653246

[4] Baiocchi GL Giacopuzzi S Marrelli D Reim D Piessen G Matos Da Costa P International consensus on a complications list after gastrectomy for cancer. Gastric Cancer. (2019) 22(1):172–89. 10.1007/s10120-018-0839-5 29846827

[5] Cozzaglio L Coladonato M Biffi R Coniglio A Corso V Dionigi P Duodenal fistula after elective gastrectomy for malignant disease. J Gastrointest Surg. (2010) 14(5):805–11. 10.1007/s11605-010-1166-2 20143272

[6] Cozzaglio L Giovenzana M Biffi R Cobianchi L Coniglio A Framarini M Surgical management of duodenal stump fistula after elective gastrectomy for malignancy: an Italian retrospective multicenter study. Gastric Cancer. (2016) 19(1):273–9. 10.1007/s10120-014-0445-0 25491774

[7] Patricia YPC Kevin WKF Yee LF Jing FK Kylie S Kee LS. Duodenal stump leakage. Lessons to learn from a large-scale 15-year cohort study. Am J Surg. (2020) 220(4):976–81. 10.1016/j.amjsurg.2020.02.042 32171473

[8] Lee KG Lee HJ Yang JY Oh SY Bard S Suh YS Risk factors associated with complication following gastrectomy for gastric cancer: retrospective analysis of prospectively collected data based on the clavien-dindo system. J Gastrointest Surg. (2014) 18(7):1269–77. 10.1007/s11605-014-2525-1 24820136

[9] Lu S Yan M Li C Yan C Zhu Z Lu W. Machine-learning-assisted prediction of surgical outcomes in patients undergoing gastrectomy. Chin J Cancer Res. (2019) 31(5):797–805. 10.21147/j.issn.1000-9604.2019.05.09 31814683 PMC6856706

[10] Paik HJ Lee SH Choi CI Kim DH Jeon TY Kim DH Duodenal stump fistula after gastrectomy for gastric cancer: risk factors, prevention, and management. Ann Surg Treat Res. (2016) 90(3):157–63. 10.4174/astr.2016.90.3.157 26942159 PMC4773460

[11] Gu L Zhang K Shen Z Wang X Zhu H Pan J Risk factors for duodenal stump leakage after laparoscopic gastrectomy for gastric cancer. J Gastric Cancer. (2020) 20(1):81–94. 10.5230/jgc.2020.20.e4 32269847 PMC7105415

[12] Kibbe W Klemm J Quackenbush J. Cancer informatics: new tools for a data-driven age in cancer research. Cancer Res. (2017) 77(21):e1–2. 10.1158/0008-5472.CAN-17-2212 29092926

[13] Shapey IM Sultan M. Machine learning for prediction of postoperative complications after hepato-biliary and pancreatic surgery. Artif Intell Surg. (2023) 3(1):1–13. 10.20517/ais.2022.31

[14] Ley C Martin RK Pareek A Groll A Seil R Tischer T. Machine learning and conventional statistics: making sense of the differences. Knee Surg Sports Traumatol Arthrosc. (2022) 30(3):753–7. 10.1007/s00167-022-06896-6 35106604

[15] Buuren S Groothuis-Oudshoorn K. Mice: multivariate imputation by chained equations inR. J Stat Softw. (2011) 45(3):1–67. 10.18637/jss.v045.i03

[16] Efendi A Effrihan E. A simulation study on Bayesian ridge regression models for several collinearity levels. AIP Conf Proc. (2017) 1913(1):1–6. 10.1063/1.5016665

[17] Hearst MA Dumais ST Osuna E Platt J Scholkopf B. Support vector machines. IEEE Intell Syst. (1988) 13(4):18–28. 10.1109/5254.708428

[18] Chen T Guestrin C. XGBoost. Proceedings of the 22nd ACM SIGKDD International Conference on Knowledge Discovery and Data Mining; 13 August 2016; San Francisco, California, USA (2016). p. 785–94

[19] Srivastava N Hinton G Krizhevsky A Sutskever I Salakhutdinov R. Dropout: a simple way to prevent neural networks from overfitting. J Mach Learn Res. (2014) 15(1):1929–58.

[20] Ioffe S Szegedy C. Batch normalization: accelerating deep network training by reducing internal covariate shift. In Proceedings of the 32nd International Conference on Machine Learning (ICML) (2015).

[21] Ahr H. Applied Logistic Regression. New Jersey: John Wiley & Sons, Inc. (2013).

[22] Fernández-Delgado M Cernadas E Barro S Amorim D. Do we need hundreds of classifiers to solve real world classification problems? J Mach Learn Res. (2014) 15:3133–81.

[23] Wu J Li Y Ma Y. Comparison of XGBoost and the neural network model on the class-balanced datasets. 2021 IEEE 3rd International Conference on Frontiers Technology of Information and Computer (ICFTIC) (2021). p. 457–61

[24] Dong S Wang P Abbas K. A survey on deep learning and its applications. Comput Sci Rev. (2021):40. 10.1016/j.cosrev.2021.100379

[25] Hussain SN Aljahdali S AhmedK A. Comparative prediction performance with support vector machine and random forest classification techniques. Int J Comput Appl. (2013) 69(11):12–6. 10.5120/11885-7922

[26] Sidey-Gibbons JAM Sidey-Gibbons CJ. Machine learning in medicine: a practical introduction. BMC Med Res Methodol. (2019) 19(1):1–18. 10.1186/s12874-019-0681-4 30890124 PMC6425557

[27] Bzdok D. Classical statistics and statistical learning in imaging neuroscience. Front Neurosci. (2017) 11:543. 10.3389/fnins.2017.00543 29056896 PMC5635056

[28] Bzdok D Krzywinski M Altman N. Points of significance: machine learning: a primer. Nat Methods. (2017) 14(12):1119–20. 10.1038/nmeth.4526 29664466 PMC5905345

[29] Hong QQ Yan S Zhao YL Fan L Yang L Zhang WB Machine learning identifies the risk of complications after laparoscopic radical gastrectomy for gastric cancer. World J Gastroenterol. (2024) 30(1):79–90. 10.3748/wjg.v30.i1.79 38293327 PMC10823896

[30] Shao S Liu L Zhao Y Mu L Lu Q Qin J. Application of machine learning for predicting anastomotic leakage in patients with gastric adenocarcinoma who received total or proximal gastrectomy. J Pers Med. (2021) 11(8):1–9. 10.3390/jpm11080748 PMC8400241 34442391

[31] Chen R-C Dewi C Huang S-W Caraka RE. Selecting critical features for data classification based on machine learning methods. J Big Data. (2020) 7(1):1–26. 10.1186/s40537-020-00327-4

[32] Grinsztajn L Oyallon E Varoquaux G. Why do tree-based models still outperform deep learning on typical tabular data? Adv Neural Inf Process Syst. (2022) (35):507–20.

[33] Shwartz-Ziv R Armon A. Tabular data: deep learning is not all you need. Inf Fusion. (2022) 81:84–90. 10.1016/j.inffus.2021.11.011

[34] Fernando KRM Tsokos CP. Dynamically weighted balanced loss: class imbalanced learning and confidence calibration of deep neural networks. IEEE Trans Neural Netw Learn Syst. (2022) 33(7):2940–51. 10.1109/TNNLS.2020.3047335 33444149

[35] Zizzo M Ugoletti L Manzini L Castro Ruiz C Nita GE Zanelli M Management of duodenal stump fistula after gastrectomy for malignant disease: a systematic review of the literature. BMC Surg. (2019) 19(1):55. 10.1186/s12893-019-0520-x 31138190 PMC6540539

[36] Orsenigo E Bissolati M Socci C Chiari D Muffatti F Nifosi J Duodenal stump fistula after gastric surgery for malignancies: a retrospective analysis of risk factors in a single centre experience. Gastric Cancer. (2014) 17(4):733–44. 10.1007/s10120-013-0327-x 24399492

[37] Witte MB Barbul A. Repair of full-thickness bowel injury. Crit Care Med. (2003) 31(8 Suppl):S538–46. 10.1097/01.CCM.0000081436.09826.A4 12907884

[38] Verhofstad MH Lange WP van der Laak JA Verhofstad AA Hendriks T. Microscopic analysis of anastomotic healing in the intestine of normal and diabetic rats. Dis Colon Rectum. (2001) 44(3):423–31. 10.1007/BF02234744 11289291

[39] Dasari N Jiang A Skochdopole A Chung J Reece EM Vorstenbosch J Updates in diabetic wound healing, inflammation, and scarring. Semin Plast Surg. (2021) 35(3):153–8. 10.1055/s-0041-1731460 34526862 PMC8432997

[40] Azzolina D Baldi I Barbati G Berchialla P Bottigliengo D Bucci A Machine learning in clinical and epidemiological research: isn't it time for biostatisticians to work on it? Epidemiol Biostat Public Health. (2022) 16(4):1–3. 10.2427/13245

[41] Stam WT Goedknegt LK Ingwersen EW Schoonmade LJ Bruns ERJ Daams F. The prediction of surgical complications using artificial intelligence in patients undergoing major abdominal surgery: a systematic review. Surgery. (2022) 171(4):1014–21. 10.1016/j.surg.2021.10.002 34801265

[42] Ng AY. Feature selection, L1 vs. L2 regularization, and rotational invariance. Twenty-first International Conference on Machine Learning - ICML ‘04: ACM Press (2004).

[43] Hua J Xiong Z Lowey J Suh E Dougherty ER. Optimal number of features as a function of sample size for various classification rules. Bioinformatics. (2005) 21(8):1509–15. 10.1093/bioinformatics/bti171 15572470

[44] Weindelmayer J Mengardo V Ascari F Baiocchi GL Casadei R De Palma GD Prophylactic drain placement and postoperative invasive procedures after gastrectomy: the abdominal drain after gastrectomy (ADIGE) randomized clinical trial. JAMA Surg. (2025) 160(2):135–43. 10.1001/jamasurg.2024.5227 39602143 PMC11822533

[45] Lim SY Kang JH Jung MR Ryu SY Jeong O. Abdominal drainage in the prevention and management of major intra-abdominal complications after total gastrectomy for gastric carcinoma. J Gastric Cancer. (2020) 20(4):376. 10.5230/jgc.2020.20.e32 33425439 PMC7781750

[46] Liu X Lei S Wei Q Wang Y Liang H Chen L. Machine learning-based correlation study between perioperative immunonutritional Index and postoperative anastomotic leakage in patients with gastric cancer. Int J Med Sci. (2022) 19(7):1173–83. 10.7150/ijms.72195 35919820 PMC9339417

[47] Lee S-H Kim KH Choi CW Kim SJ Kim D-H Choi CI Reduction rate of C-reactive protein as an early predictor of postoperative complications and a reliable discharge indicator after gastrectomy for gastric cancer. Ann Surg Treat Res. (2019) 97(2):65. 10.4174/astr.2019.97.2.65 31384611 PMC6669129

